# Predictive functional analysis reveals inferred features unique to cervicovaginal microbiota of African women with bacterial vaginosis and high-risk human papillomavirus infection

**DOI:** 10.1371/journal.pone.0253218

**Published:** 2021-06-18

**Authors:** Harris Onywera, Joseph Anejo-Okopi, Lamech M. Mwapagha, Javan Okendo, Anna-Lise Williamson

**Affiliations:** 1 Institute of Infectious Disease and Molecular Medicine, University of Cape Town, Cape Town, South Africa; 2 Faculty of Health Sciences, Division of Medical Virology, Department of Pathology, University of Cape Town, Cape Town, South Africa; 3 Research, Innovations, and Academics Unit, Tunacare Services Health Providers Limited, Nairobi, Kenya; 4 Department of Microbiology, University of Jos, Jos, Nigeria; 5 AIDS Prevention Initiative in Nigeria, Jos University Teaching Hospital, Jos, Nigeria; 6 Faculty of Health and Applied Sciences, Department of Natural and Applied Sciences, Namibia University of Science and Technology, Windhoek, Namibia; 7 Division of Chemical and Systems Biology, Faculty of Health Sciences, Department of Integrative Biomedical Sciences, University of Cape Town, Cape Town, South Africa; 8 Centre for Research in Therapeutic Sciences (CREATES), Strathmore University, Nairobi, Kenya; 9 SAMRC Gynaecological Cancer Research Centre, University of Cape Town, Cape Town, South Africa; Universidade Catolica Portuguesa, PORTUGAL

## Abstract

Mounting evidence suggests that *Lactobacillus* species may not necessarily be the *sine qua non* of healthy cervicovaginal microbiota (CVM), especially among reproductive-age African women. A majority of African women have high-diversity non-*Lactobacillus*-dominated CVM whose bacterial functions remain poorly characterized. Functional profiling of the CVM is vital for investigating human host-microbiota interactions in health and disease. Here, we investigated the functional potential of *L*. *iners*-dominated and high-diversity non-*Lactobacillus*-dominated CVM of 75 African women with and without bacterial vaginosis (BV) and high-risk human papillomavirus (HR-HPV) infection. Functional contents were predicted using PICRUSt. Microbial taxonomic diversity, BV, and HR-HPV infection statuses were correlated with the inferred functional composition of the CVM. Differentially abundant inferred functional categories were identified using linear discriminant analysis (LDA) effect size (LEfSe) (p-value <0.05 and logarithmic LDA score >2.0). Of the 75 women, 56 (74.7%), 35 (46.7%), and 29 (38.7%) had high-diversity non-*Lactobacillus*-dominated CVM, BV, and HR-HPV infection, respectively. Alpha diversity of the inferred functional contents (as measured by Shannon diversity index) was significantly higher in women with high-diversity non-*Lactobacillus*-dominated CVM and BV than their respective counterparts (H statistic ≥11.5, q-value <0.001). Ordination of the predicted functional metagenome content (using Bray-Curtis distances) showed that the samples segregated according to the extent of microbial taxonomic diversity and BV (pseudo-F statistic ≥19.6, q-value = 0.001) but not HR-HPV status (pseudo-F statistic = 1.7, q-value = 0.159). LEfSe analysis of the inferred functional categories revealed that transport systems (including ABC transporters) and transcription factors were enriched in high-diversity CVM. Interestingly, transcription factors and sporulation functional categories were uniquely associated with high-diversity CVM, BV, and HR-HPV infection. Our predictive functional analysis reveals features unique to high-diversity CVM, BV and HR-HPV infections. Such features may represent important biomarkers of BV and HR-HPV infection. Our findings require proof-of-concept functional studies to examine the relevance of these potential biomarkers in women’s reproductive health and disease.

## Introduction

A healthy cervicovaginal microbiota (CVM) of reproductive-age women is regarded as one that is colonized predominantly by a single or multiple *Lactobacillus* species, notably *L*. *crispatus*, *L*. *gasseri*, *L*. *iners*, or *L*. *jensenii* [1]. However, molecular studies have challenged this long-held concept of a healthy CVM following the observations of non-*Lactobacillus*-dominated CVM, often with high bacterial diversity, among some healthy women. A series of studies have observed that the patterns of CVM vary markedly among women from different ethnic/racial background, with high-diversity CVM being not uncommon among women of African descent [2,3], including African Surinamese, Ghanaian [3], Nigerian [4], Kenyan [5], Rwandan [6], and South African women [7,8]. Moreover, among the few African women with *Lactobacillus*-dominated CVM, CVM with *L*. *iners* dominance is often the most prevalent [2,3,5,8]. Among the common cervicovaginal *Lactobacillus* spp., *L*. *iners* has been pointed out to be the least stable and least protective; hence, can facilitate transition between bacterial vaginosis (BV) and non-BV (other *Lactobacillus*-dominated) states [1]. Women colonized with *L*. *iners* are more likely to be predisposed to BV relative to women colonized with *L*. *crispatus* [9].

The differences in CVM composition within African women and between African women and women from other ethnicities/races could be attributed to human host genetic background [10], sociodemographic, sexual behavioural, and clinical factors [3,5,8,10,11]. Whereas lack of appreciable numbers of *Lactobacillus* spp. does not necessarily reflect an unhealthy CVM [12] and has been posited to be either intermediate or variant states of health [8], such CVM with high microbial diversity have been associated with urogenital syndromes, infections, and diseases that include BV [8,13], cervical intraepithelial neoplasia (CIN) [14], and sexually transmitted infections (STIs) such as human immunodeficiency virus (HIV) and human papillomavirus (HPV) [6]. BV, STIs, and cervical cancer cases are more widespread in sub-Saharan Africa compared to other world regions [15–17].

Most studies have focussed on exploring the structure (composition and diversity) of the CVM of women with and without genital syndromes, infections, and diseases. These studies have shown that the structure of the CVM of such women varies according to cervicovaginal conditions [13,14]. A caveat of taxonomic approach of studying CVM is that it does not provide insight into functional and metabolic contents of the bacteria. Thus, to better understand the effects of CVM on women’s reproductive health, the taxonomic profile must be linked with the functional profile of the CVM. In other words, it is not the presence of a bacterium in the host that matters, but what it does. Studies examining the linkages between the taxonomic and functional profiles of CVM have at least suggested that human host-microbiota interactions affect physiology of the bacteria and host. Such alterations may be in the bacterial metabolic pathways and virulence potential [18–21] or metabolic, protein compositional, cellular component, and immunological changes in the host [18,22]. Some of these changes, for example, proteomic signatures associated with highly diverse *Gardnerella vaginalis*-dominated CVM, can consequently alter the cervicovaginal epithelial barrier [18], thereby decreasing its limit to infections. Approaches that have been utilized to estimate the functional and metabolic contents of microbiota include metagenomics [11,20,23–25], metaproteomics [18], and computational analysis, e.g., phylogenetic investigation of communities by reconstruction of unobserved states (PICRUSt) [26] and Tax4Fun [27] using 16S rRNA marker gene sequences and databases such as Kyoto Encyclopedia of Genes and Genomes (KEGG) and Cluster of Orthologous Groups (COG).

PICRUSt is a validated bioinformatics tool that has been widely used for predicting the functional contents of microbiota of different environmental sites. However, it is worth noting that there are less than a handful CVM studies that have used PICRUSt to infer the functional potential of bacteria [10,21,28]. A CVM study that used PICRUSt to examine the metagenome functions in 77 Taiwanese women found 90 differentially abundant inferred KEGG functional categories between women with and without BV [21]. Bacterial invasion of epithelial cells, bacterial chemotaxis, and bacterial motility proteins were among the inferred functional categories that were more enriched in women with BV relative to women without BV [21]. Bacterial chemotaxis and motility are critical for successful niche colonization and disease development [29]. Thus, PICRUSt can be used to mine meaningful functional metagenomic capacity of CVM in women with cervicovaginal syndromes, infections, and diseases.

The CVM of women in sub-Saharan Africa is becoming more known [4,6–8,30]. For example, we have consistently reported that *L*. *iners* and high-diversity non-*Lactobacillus*-dominated CVM are the most common CVM among South African women (prevalence: 22–39% and 57–64%, respectively) [8,30]. Furthermore, we are aware that sub-Saharan Africa is burdened by BV [17] and HPV [15,16] infection and that studies, including ours, though few, have associated HPV infection with lower relative abundances of *L*. *iners* concomitant with higher relative abundances of BV-associated bacteria, notably *G*. *vaginalis*, *Atopobium vaginae*, *Prevotella* sp., and *Sneathia* sp. [4,30]. However, until now, little is known about the functional composition of CVM of women of African descent, including South African women with and without BV and HPV infections. We therefore sought to use PICRUSt [26] to infer the functional metagenomic capacity of the CVM of reproductive-age South African with and without high-diversity CVM, BV, and HR-HPV infection.

## Materials and methods

### Study design and ethics approval

Endocervical samples for the present functional study of the CVM were obtained from the HPV Couples Cohort Study that investigated the concordance of genital HPV infection in heterosexually active South African couples and the impact of HIV coinfection [31]. Ethical approval for the parent and present studies were obtained from the Human Research Ethics Committee (HREC) of the University of Cape Town, South Africa (HREC references 258/2006 and 580/2014, respectively). The HPV Couples Cohort Study had recruited participants after obtaining their informed written consents to participate in the study and permit use of their samples for future studies. Two swabs were collected from the cervix during speculum examination. The first swab was for Papanicolaou (Pap) smear, which was used for microbiological diagnosis of BV according to the Bethesda criteria for reporting cervical/vaginal cytologic diagnoses [32]. Here, clue cells with coccobacilli (mostly *G*. *vaginalis*) and/or noticeable absence of lactobacilli on wet microscopy were regarded as an indication of BV. The second swab, which was for HPV genotyping and CVM study, was stored in Digene specimen transport medium (Digene Corporation, Gaithersburg, MD, USA) at -80°C until nucleic acid extraction. HPV genotyping was performed using the Roche Linear Array HPV genotyping test (Roche Molecular Diagnostics, Mannheim, Germany) that detects 37 HPV genotypes: 12 oncogenic high-risk (HR), 8 probable oncogenic HR, and 17 non-oncogenic low-risk HPV types as documented elsewhere [31]. The data were analyzed anonymously.

The inclusion and exclusion criteria and the study population characteristics of the 87 reproductive-age (18–44 years) HIV-seronegative women included in this study are detailed elsewhere [30]. All the women were neither menstruating nor pregnant at the time of sample collection. Thirty eight (43.7%) and 30 (34.5%) of the women were positive for BV and HR-HPV infection, respectively. A majority (75.6%) of the women had normal cervical cytology. About one-third (32.2%) of the women were smoking cigarettes at the time of study.

Comparison of baseline characteristics of the women according to HR-HPV infection status indicated that younger age was associated with HR-HPV positivity (28.0 (24.5–35.0) versus 35.0 (27.8–41.0) years; p-value = 0.015). Cervical cytology was statistically different between HR-HPV-negative versus HR-HPV positive group (p-value = 0.028), with HR-HPV-positive women being less likely to have normal cervical cytology compared to HR-HPV-negative women (57.7% versus 84.1%, odds ratio: 0.3 (95% confidence interval, CI [0.1–0.8]); p-value = 0.015). None of the variables statistically differed between women with and without BV.

### Genomic DNA extraction, Illumina MiSeq sequencing of barcoded 16S rRNA gene amplicons, and sequence data analysis

Genomic DNA extraction, amplification, metabarcoding library preparation, paired-end sequencing, and data analysis were performed as previously described [30]. The hypervariable V3-V4 region of the 16S rRNA gene were targeted using universal bacterial primers PCR 319F (5’-CCTACGGGNGGCWGCAG-3’) and 806R (5’-GACTACHVGGGTATCTAATCC-3’). PCR and sequencing controls were run concomitantly with the 87 samples. These included i) nuclease free water as a negative control, ii) Digene specimen transport medium as an extraction control to check for possible contaminants, and iii) two mock bacterial communities, HM-782D and HM-783D (BEI Resources, Manassas, VA, USA), comprising of genomic DNA from 20 bacterial strains, as positive controls. HM-782D contained equimolar concentrations (100,000 rRNA operons per organism per μl) of each of the 20 bacteria. In HM-783D, the concentrations of these bacteria were staggered (ranging from 1,000 to 1,000,000 rRNA operons per organism per μl). A dual-indexing approach amplification approach was used to prepare 16S rRNA gene amplicon libraries, which were later purified using Agencourt AMPure XP System (Beckman Coulter, Beverly, MA, USA). Libraries were then pooled in equimolar ratios and sequenced on an Illumina MiSeq by Macrogen Inc. (Seoul, South Korea).

QIIME (quantitative insights into microbial ecology) v1.8.0 [33] and UPARSE (usearch8.0.1616) [34] were used to analyse bacterial community sequence data. The steps (including clustering of operational taxonomic units (OTUs), taxonomic classification of representative sequences and diversity estimations) and parameters used in this bioinformatics analysis are published in details elsewhere [30]. Briefly, OTU clustering was performed at 97% sequence similarity threshold, taxonomy assignment with the RDP Naïve Bayesian Classifier [35] using Greengenes database (gg13_8 Release) [36]. For the taxonomic profile, OTU clustering was achieved using UPARSE-OTU method that is able to perform both closed-reference and *de novo* OTU picking using a greedy clustering algorithm [34]. Beta diversity estimates of the taxonomic profile were performed using a rarefied OTU table (12,161 reads per sample).

### Identification of community state types (CSTs)

All our analyses were based on high-quality filtered sequence data. This was confirmed by the observations of minimal to absent false positives and negatives (in terms of reads) in our dataset, including controls (results not shown), as reported elsewhere [30]. Moreover, we performed a non-intuitive OTU filtering based on the proportion of the most abundant false positive (*L*. *iners*: 0.0222%) in the even mock community control (HM-782D). Here, we assumed that any amplification and sequencing error in our dataset was homogeneous across the samples. So, using QIIME’s “filter_otus_from_otu_table.py” script, we used the argument “—min_count_fraction” to filter OTUs from the OTU table based on the 0.0222% threshold. The argument “—min_count_fraction” is defined as the fraction of the total observation (sequence) count to apply as the minimum total observation count of an OTU for it to be retained in the OTU table (http://qiime.org/scripts/filter_otus_from_otu_table.html).

For the identification of the CSTs, we relied on an unrarefied OTU table (unlike in beta diversity estimations). Average neighbour linkage unsupervised clustering of the samples was performed (based on Bray-Curtis dissimilarity index) as previously documented [30] in order to identify the groups (CSTs) of bacterial communities based on clustering patterns and relative abundance of bacterial taxa. The CVM of the 87 South African women were grouped according to community composition and relative abundance. This included all the *Lactobacillus* spp. and bacteria with ≥0.33% relative abundance. Alpha diversity estimations of the CSTs were re-examined using rank abundance curves of the OTUs.

### Identification of differentially abundant taxa between the most prevalent CSTs

Further analyses to detect differentially abundant bacterial taxa (in the unrarefied OTU table) between the most prevalent CSTs were performed by statistical analyses of metagenomic (and other) profiles (STAMP) v2.1.3 [37]. The White’s non-parametric t-test (two-sided type) [38] was used for computation. The confidence interval method used was the difference between mean proportions (DP): bootstrap. The thresholds for p-value and effect size were 0.05 and 1.0, respectively. The p-values were corrected to false discovery rate (FDR) q-values (with 0.05 as the threshold for significance) using the Benjamini-Hochberg procedure.

### Prediction of functional profiles of the bacterial communities

PICRUSt v1.1.0 [26] was used to infer the functional metagenomic contents of each sample (in unrarefied OTU table) and to categorize the inferred functional genes counts to the KEGG pathways. These investigations were performed as described elsewhere [39], with minor modifications. Since PICRUSt performs only on closed-reference picked OTUs, we picked these OTUs with the Greengenes database (gg13_8 Release) [36] as the reference. PICRUSt accuracy across the samples was measured using weighted nearest sequenced taxon index (NSTI). NSTI score is calculated as follows: “For every OTU in a sample, the sum of branch lengths between that OTU in the Greengenes tree to the nearest tip in the tree with a sequenced genome is weighted by the relative abundance of that OTU. All OTU scores are then summed to give a single NSTI value per microbial community sample” [26]. NSTI scores, ranges from 0 to 1 and reflect the availability of reference genomes that are closely related to the most abundant microbe in one’s sample. High NSTI values mean few related references are available for the respective sample. As a result, the predictions will be of low quality. The converse is true for low NSTI values.

In order to determine whether the CVM could be grouped by their predominant inferred functional categories at level 3 KEGG Orthology (KO), we used DESeq2 v1.26.0 [40] in R v3.6.1 to perform spectral clustering of normalized log_2_-transformed raw count matrix data of the top 50 inferred functional categories with the greatest variance. These results were displayed on a heatmap generated using R package pheatmap v1.0.12 (https://CRAN.R-project.org/package=pheatmap). Associations of the identified functional clusters with BV, HR-HPV, and CST (taxonomic clusters) were computed according to Chi-square/Fisher’s exact tests (with two-tailed p-value) using GraphPad Prism v6.01 (San Diego, USA).

Alpha diversity estimates of the predicted functional contents (according to CST, BV, and HR-HPV infection statuses) were computed using Shannon diversity and Bray-Curtis dissimilarity indices, respectively, within QIIME. For beta analyses, the number of predicted functional contents in each sample was normalized to 5,921,112 across all the samples. Next, the correlations between the participant information (diversity of the CVM, BV and HR-HPV status) and inferred functional categories were studied using principal coordinate analysis (PCoA). Differences in the abundances of the predicted functional categories were analyzed and visualized using linear discriminant analysis (LDA) effect size (LEfSe) v1.0 [41] and STAMP v2.1.3 [37]. For LEfSe, the non-parametric factorial Kruskal-Wallis (KW) sum-rank test was used to calculate the predicted functional categories with significant differential abundance (p-value <0.05) with respect to the following: i) extent of high-diversity the CVM, ii) BV status, and HR-HPV infection status. The effect size of each differentially abundant functional module was estimated using LDA. Only discriminative modules with logarithmic LDA scores of >2.0 (absolute) were plotted on the histograms.

## Results

### Cervicovaginal community composition and diversity

**Fig 1:**
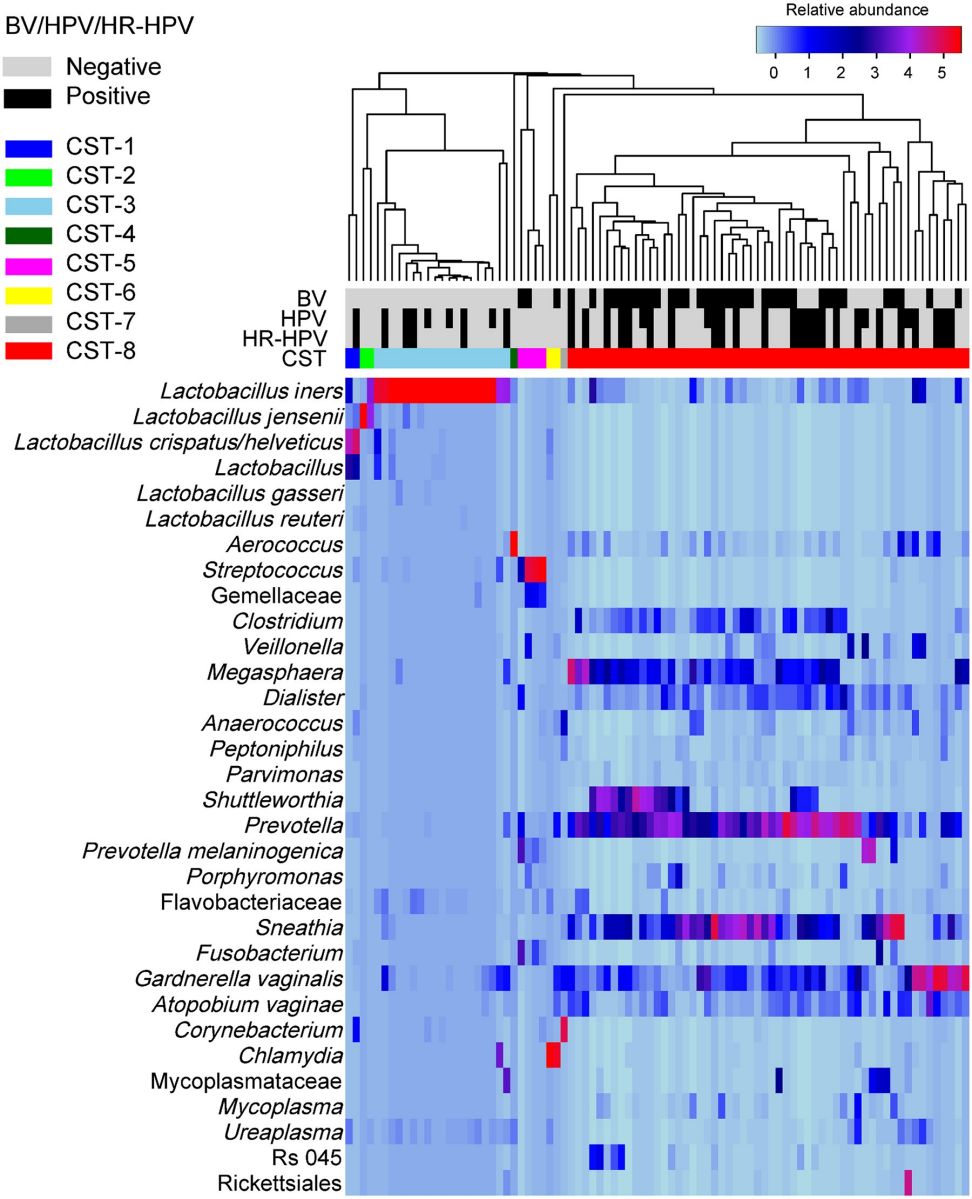
Average linkage hierarchical clustering of the cervicovaginal samples based on the composition and relative abundance of bacterial communities. The colour key of the relative abundances is indicated in the upper right corner. A scale towards blue (0) and red (5) indicates a lower and higher relative abundance, respectively. Rows represent the bacterial taxa whereas the columns represent the samples. All bacterial taxa with ≥0.33% relative abundance are shown. All *Lactobacillus* spp., regardless of their relative abundances were included in the analysis. Bacterial vaginosis (BV), human papillomavirus (HPV) and high-risk (HR)-HPV infection status as well as the community state type are indicated. Eight microbiota clusters were detected based on Bray-Curtis index.

The heatmap in Fig 1 shows the eight distinct CSTs that were detected in our cohort. In brief, 56 women (64.4%) had diverse CVM (CST-8) with predominance of BV-associated bacteria such as *G*. *vaginalis*, *A*. *vaginae*, *Dialister* sp., *Clostridium* sp., *Megasphaera* sp., and *Sneathia* sp. The other CVM were dominated by *L*. *crispatus* (CST-1: 2.3%), *L*. *jensenii* (CST-2: 2.3%), *L*. *iners* (CST-3: 21.8%), *Aerococcus* sp. (CST-4: 1.1%), *Streptococcus* sp. (CST-5: 4.6%), *Chlamydia trachomatis* (CST-6: 2.3%), or *Corynebacterium* sp. (CST-7: 1.1%). Most of the downstream analyses focussed on the most prevalent CSTs (CST-3 and CTS-8).

**Fig 2:**
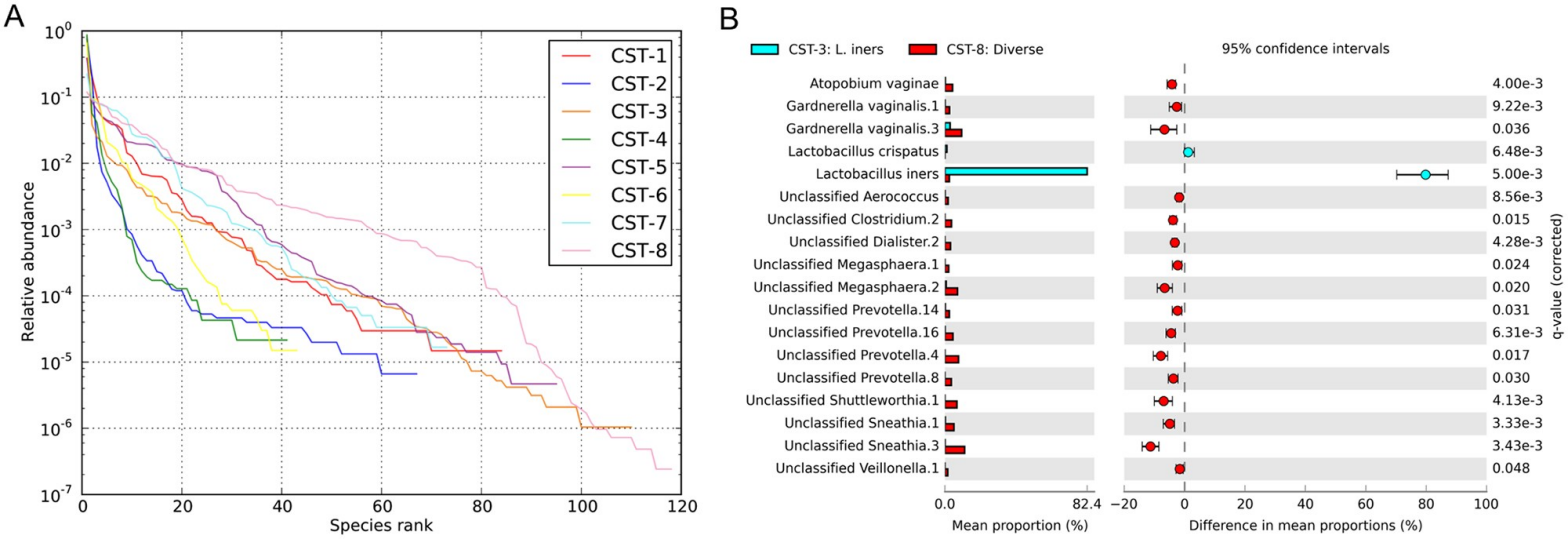
Diversity and composition of the cervicovaginal microbiota. (A) Rank abundance plots of bacterial taxa in the eight established community state types. Rank abundance plots were used to estimate the bacterial diversity by measuring richness, dominance, and evenness. Richness is the distance a curve extends along the x-axis while dominance is the y-intercept. A higher value on the y-axis depicts increased dominance; hence, low diversity. Evenness is indicated by a low slope of the curve. Therefore, in a rank abundance plot, a high dominance, more richness and evenness in a bacterial community indicate a high ecological diversity. (B) STAMP extended error bar plots depicting enriched bacterial taxa in two community state types. Only features with greater than zero difference between their percentage proportions in CST-3 and CST-8 with an effect size ≥1.0 and q-value <0.05 are shown. The statistical comparison was carried out in STAMP. The p-values (adjusted by Benjamini-Hochberg correction to account for false discovery rates), effect size and CI (0.95, DP: bootstrap) computed by White’s non-parametric t-test (two-sided type) are indicated. The difference in mean proportions and 95% CI are shown on the bar plots.

The alpha diversities of the CSTs were determined by rank abundance of the bacterial community populations (Fig 2A). For the analysis discussed hereafter, we focussed on the most prevalent CSTs, i.e., CST-3 and CST-8. The rank abundance plots showed that CST-3 and CST-8 differed in their community compositions, with CST-8 being richer, more even, and having less bacterial dominance than CST-3. Additional analysis by STAMP highlighted that 2 and 16 bacterial taxa were more enriched in CST-3 and CST-8, respectively (Fig 2B). In CST-3, *L*. *iners* and *L*. *crispatus* were in greater relative abundance than in CST-8 (p-value <0.05, q-value <0.001). In CST-8, BV-associated bacteria were more abundant than in CST-3 (p-value <0.05, q-value <0.05). Comparison of the demographic, sociobehavioural, and clinical characteristics of the women in CST-3 and CST-8 are presented in S1 Table. BV was the only significant characteristics which differed between CST-3 and CST-8 (p-value <0.0001). All the women with BV had diverse and heterogeneous CVM classified as CST-8.

### Predicted functional categories across CSTs, BV, and HR-HPV infection

To predict the functional metagenomic capacity of the CVM, we used PICRUSt [26]; a software that uses evolutionary modelling to predict the potential functional composition of bacterial communities using marker gene (such as 16S rRNA herein) sequence data and a reference database (such as KEGG herein). S1 Fig shows the per-sample weighted NSTI values that support the accuracy of PICRUSt predictions. About 70% of the samples (n = 51) had NSTI score of ≤0.15, thus indicating that the imputed functions are likely to be correlated with metagenomic data. High values imply poorly explored OTUs [26], thus indicating that the metagenomic estimates from these samples are only indicative.

**Fig 3:**
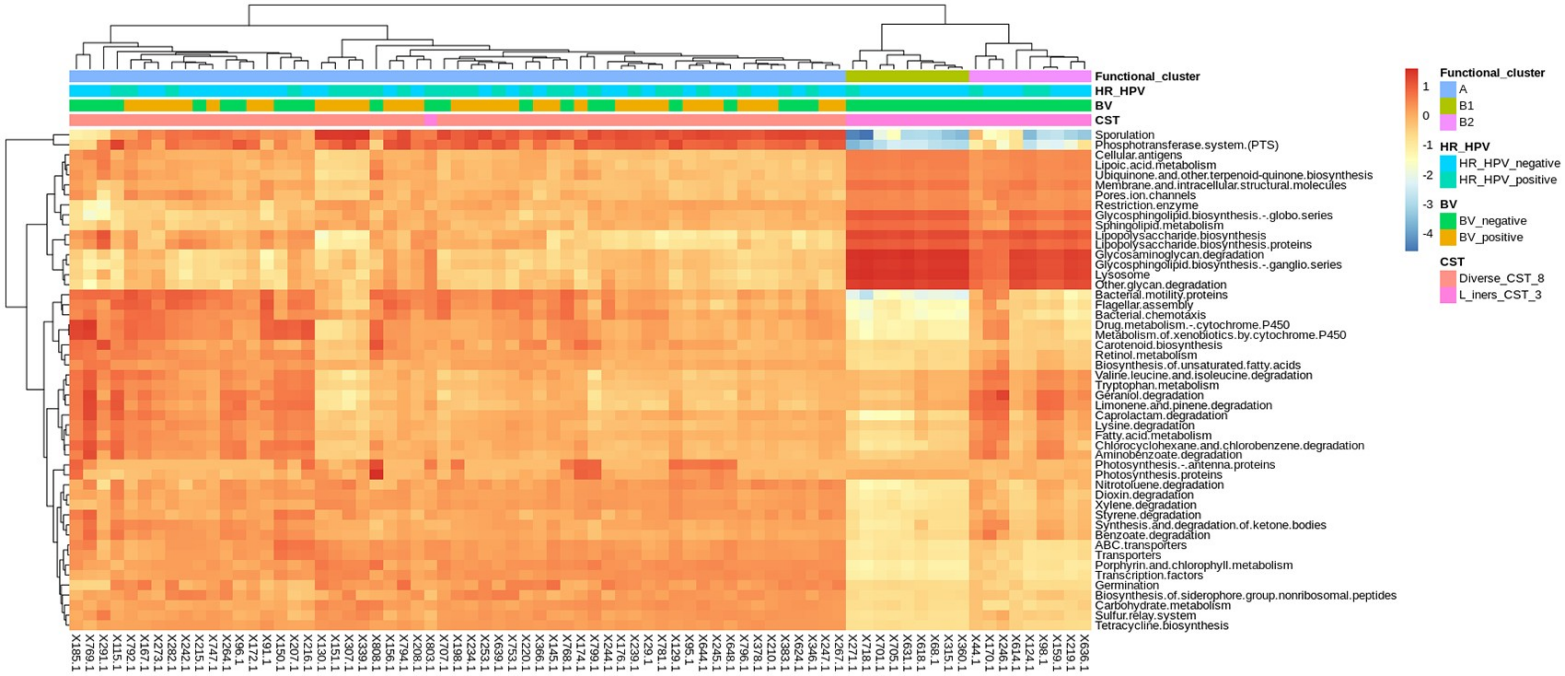
Clustering of the predicted functional categories. The spectral clustering was based on normalized log_2_-transfomred raw count matrix on the predicted functional categories with the greatest variance. The colour key of the log_2_-transformed counts is indicated in the upper right corner. Rows represent the predicted functional categories of the bacterial communities whereas the columns represent the sample identities. Only the top 50 most significant functional modules are shown. Bacterial vaginosis (BV) and high-risk human papillomavirus (HR-HPV) infection status as well as both the taxonomic and functional community state types are indicated beside the colour key. Similar to the taxonomic clusters, two functional clusters (Group-A and Group-B) using were identified.

At level 3 of the KEGG database, 264 predictive functional categories were identified. The most predominant ones included transporters (relative abundance: 6.0%), general function prediction only (3.5%), ATP-binding cassette (ABC) transporters (3.0%), DNA repair and recombination proteins (2.7%), and ribosome (2.3%). We represented top 50 predicted functional categories with the greatest variance using a heatmap (Fig 3). Based on the spectral clustering, we noted two distinct clusters of the predicted functional contents: Group-A (prevalence: 76.0%) and Group-B (24.0%). Compared to Group-B, Group-A was more heterogeneous and diverse, and had higher abundances of sporulation, phosphotransferase system (PTS), transporters, bacterial motility proteins, bacterial chemotaxis, and flagellar assembly as well as lower abundances of glycosaminoglycan degradation, glycosphingolipid biosynthesis (ganglio series), and lysosome to mention a few. Group-B could further be classified into equal halves: Group-B1 and Group-B2. Group-B1’s unique feature was the lower abundance of bacterial motility proteins and lower diversity compared to Group-B2. Group-A was negatively and positively associated with prevalent BV and CST-3, respectively; whereas Group-B was positively associated with prevalent BV and CST-8 (p-value <0.0001).

**Fig 4:**
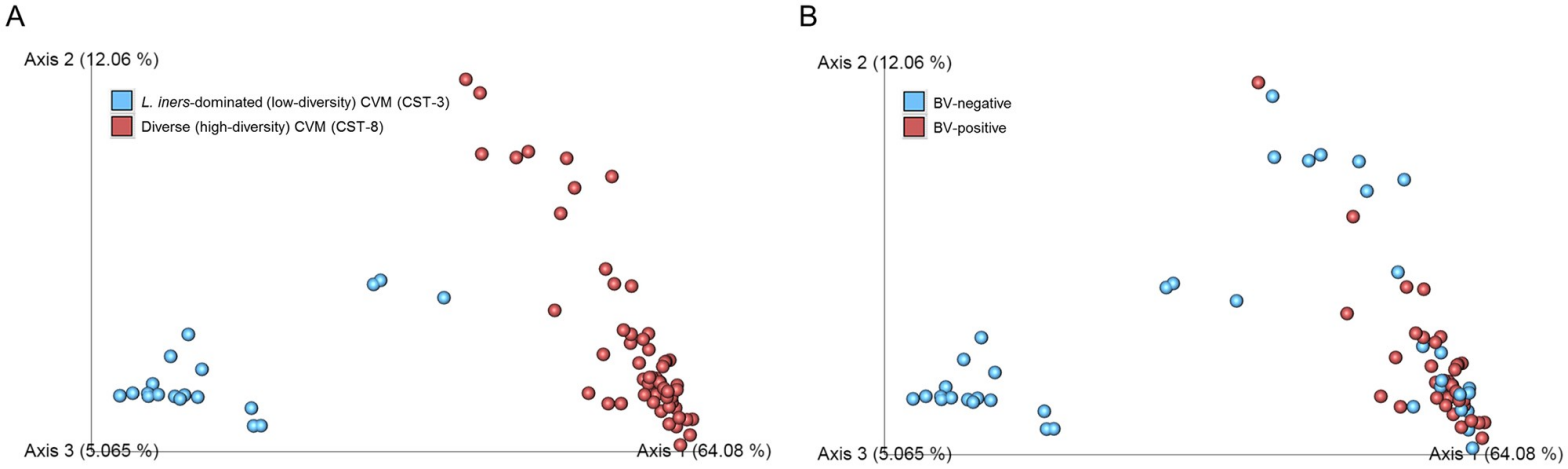
Beta diversity metric with principal component analysis (PCoA) clustering of the predicted metagenomic content. (A) PCoA according to community state type (CST, Maya blue: CST-3 and Indian red: CST-8). (B) PCoA according to BV status (Maya blue: BV-negative and Indian red: BV-positive). Each solid dot represents one cervicovaginal sample. The first three PCoA axes and the percentage variation explained by each indicated are shown (Axis 1: 64.08%, Axis 2: 12.06%, and Axis 3: 5.065%).

To estimate the diversity in the inferred functional composition of the CVM according to CST, BV, and HR-HPV-status, we used alpha and beta diversity measures as computed by Shannon diversity and Bray-Curtis dissimilarity index, respectively. We noted that the alpha diversity of the inferred functional contents was significantly higher in women with high-diversity non-*Lactobacillus*-dominated CVM (H statistic = 35.9, q-value <0.0001) and BV (H statistic = 11.5, q-value <0.0007) than their respective counterparts. Women with and without HR-HPV infection did not have statistically different in alpha diversity (H statistic = 0.38, q-value = 0.535). We corroborated the associations of the functional clusters with participant variables (CST, BV, and HR-HPV infection status) using PCoA of the inferred functional categories. We found that the inferred functional categories separated according to the taxonomic diversity of the CVM (pseudo-F statistic = 104.3, q-value = 0.001; Fig 4A) and BV status (pseudo-F statistic = 19.6, q-value = 0.001; Fig 4B) but not HR-HPV infection (pseudo-F statistic = 1.7, q-value = 0.159; S2 Fig).

**Fig 5:**
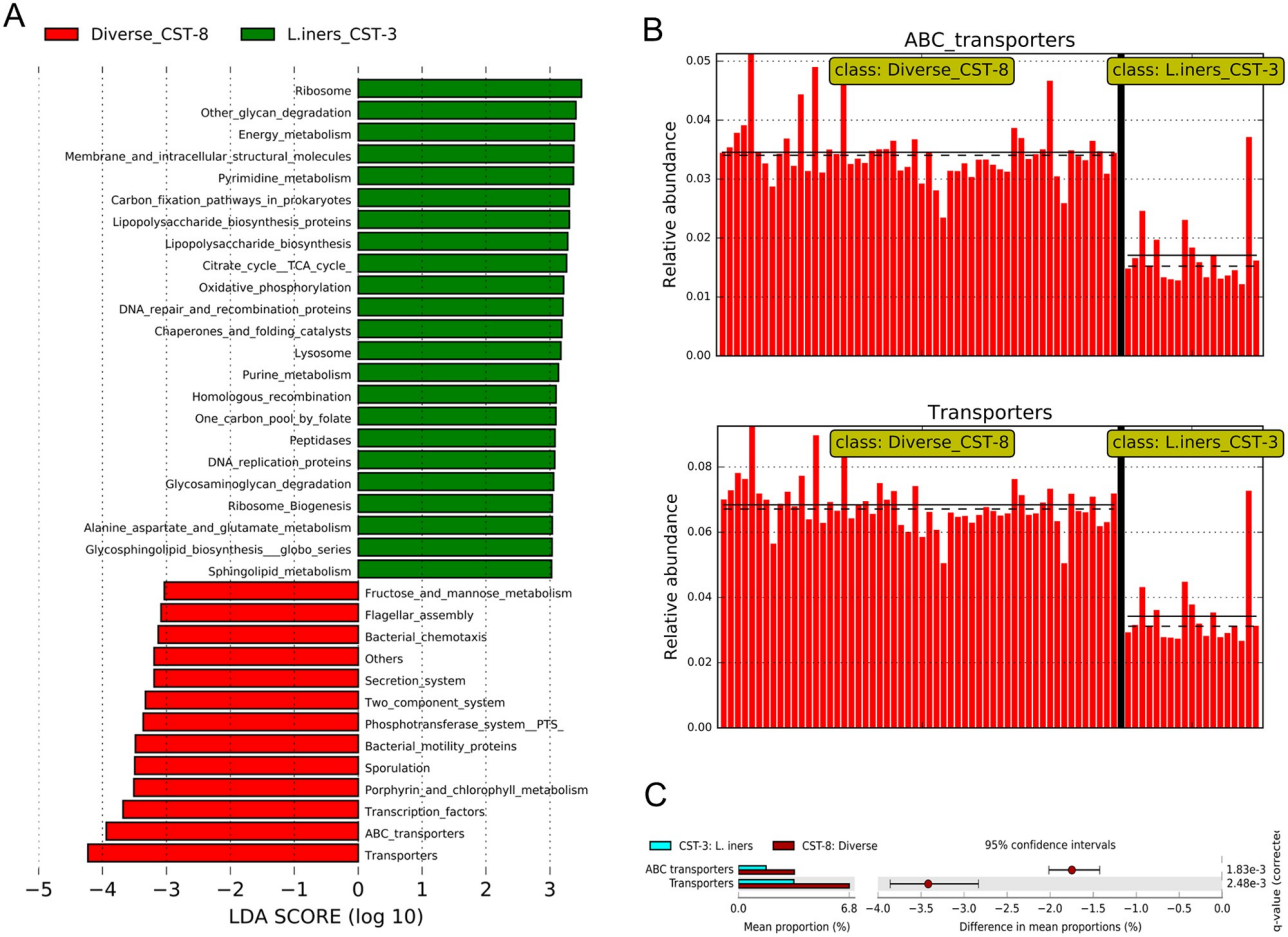
Differentially abundant inferred KEGG functional categories in diverse CVM (CST-8) and *L*. *iners*-dominated CVM (CST-3). (A) LEfSe histogram of the differentially functional modules at a logarithmic LDA score >3.0 (absolute). (B) LEfSe histograms of relative abundances of the KEGG category “transporters” (including ABC transporters) in CST-3 and CST-8. Each bar plot represents the relative abundance of the predicted transporter in each sample. The mean and median relative abundances of these transporters are shown by solid and dashed lines, respectively. (C) Extended error bar plots depicting enriched KEGG-inferred functional categories in CST-3 and CST-8. Only functional modules identified by PICRUSt with greater than zero difference between their percentage proportions in CST-3 and CST-8 with an effect size ≥1.0 and q-value ≤0.05 are shown. The statistical comparison was carried out in STAMP. The p-values (adjusted by Benjamini-Hochberg correction to account for false discovery rates), effect size and confidence intervals (0.95, DP: bootstrap) computed by White’s non-parametric t-test (two-sided type) are shown. The difference in mean proportions and 95% confidence intervals are shown on the bar plots.

Next, we performed LEfSe analysis to test whether the CVM of women with *L*. *iners* dominated CVM (CST-3) and BV-associated CVM (CST-8) have differentially abundant bacterial functions. A total of 169 inferred KEGG functional categories significantly differed between CST-3 and CST-8 (p-value <0.05, logarithmic LDA score >2.0). At a logarithmic LDA score of >3.0, 36 inferred functional categories differentially abundant as indicated in Fig 5A. Twenty three and 13 inferred functional categories were enriched in CST-3 and CST-8, respectively. The relative abundances of transporters and ABC transporters were strongly associated with CST-8. Comparison of these inferred functional categories in CST-3 versus CST-8 is shown in Fig 5B. Additional modules identified by LEfSe analysis to be enriched in CST-8 included bacterial chemotaxis, bacterial motility, and flagellar assembly. CST-3 was enriched with functional categories such as ribosomes, ribosome biogenesis, other glycan degradation, energy metabolism, citrate cycle, and DNA replication proteins to cite a few. Using STAMP [37], we found that only transporters (including ABC transporters) were differentially abundant between CST-3 and CST-8 (q-value <0.003), Fig 5C.

**Fig 6:**
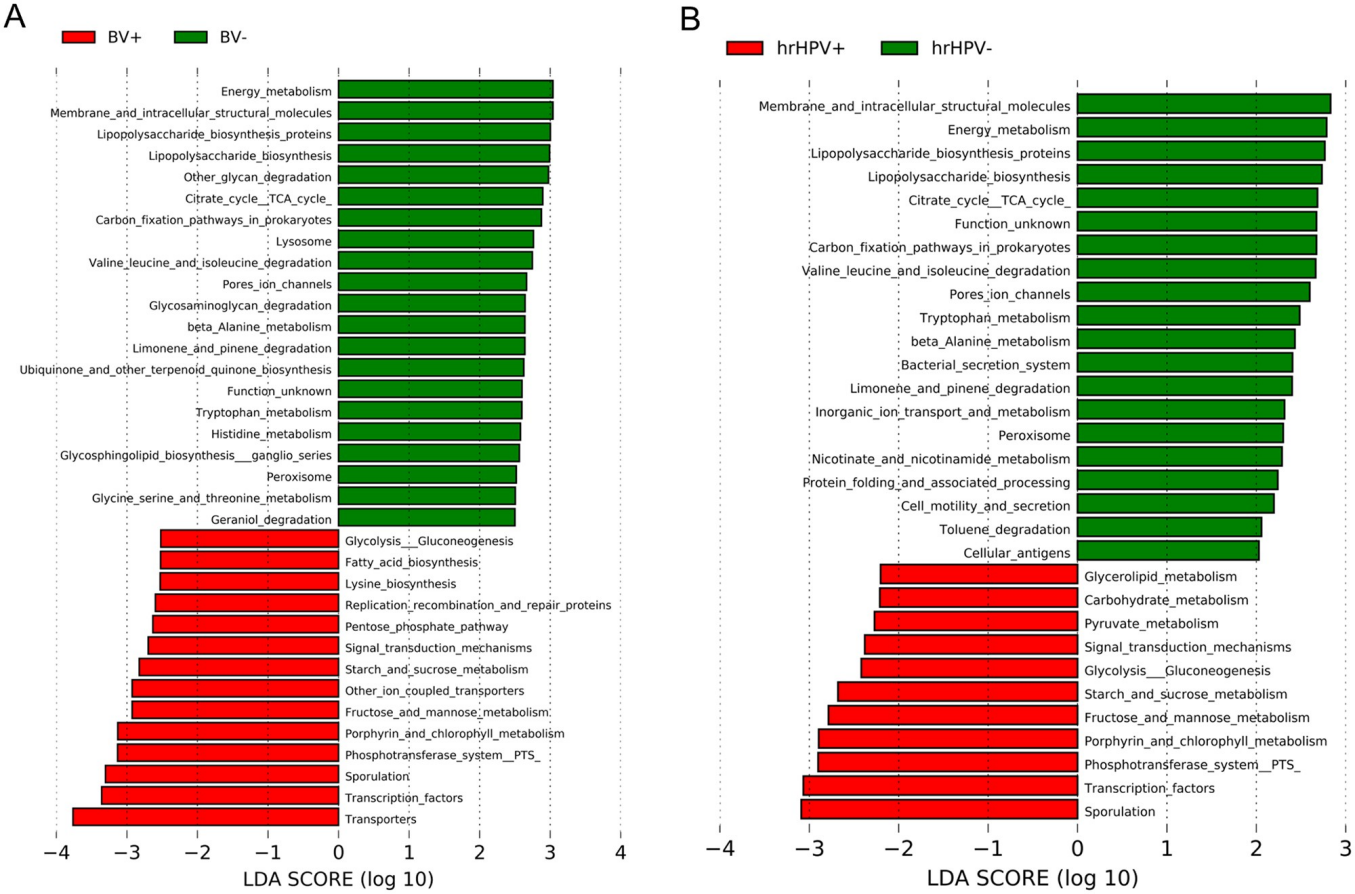
LEfSe histogram of the differentially enriched inferred KEGG functional categories. (A) Differentially abundant modules in women with and without bacterial vaginosis (BV). For better visualization, only modules with logarithmic LDA scores >2.5 (absolute values) are shown. (B) Differentially abundant modules in women with and without high-risk human papillomavirus (HR-HPV) infection. Only modules with logarithmic LDA scores >2.0 (absolute values) are shown.

In the context of BV, there were 78 predictive functional categories between the CVM of women with and without BV. Thirty five inferred KEGG functional categories differed significantly (p-value <0.05, logarithmic LDA score >2.5) between the groups (Fig 6A), with 14 modules being enriched in the CVM of women with BV. The functional categories in the CVM of women with BV included transporters, transcriptional factors, sporulation, fructose and mannose metabolism, porphyrin metabolism, phosphotransferase system, etc. In the CVM of women without BV, these comprised energy metabolism, membrane and intracellular structural proteins, lipopolysaccharide (LPS) biosynthesis, other glycan degradation, citrate cycle, and pore ion channels. Based on STAMP results (results not shown), transporters were the only predictive functional categories that differed significantly between women with and without BV (q-value = 0.005).

Lastly, we compared the differences in relative abundances of the inferred functional categories between samples of women with and without HR-HPV infection. Samples from women with HR-HPV infection had 11 predictive functional categories (e.g., sporulation and transcriptional factors) while those from women without HR-HPV infection had 20 categories (e.g., membrane and intracellular structural molecules and inorganic ion transport), p-value <0.05, logarithmic LDA score >2.0 (Fig 6B).

It is interesting to point out that several inferred functional categories were significantly differentially abundant in all three comparisons (CST, BV, and HR-HPV infection). Serving as examples are sporulation and transcription factors, which were enriched in the high diversity CVM (CST-8) as well as CVM of women with BV and HR-HPV infection. In contrast, the CVM of women without BV and HR-HPV infection and with *L*. *iners* dominance were enriched with membrane and intracellular structural molecules, LPS biosynthesis, amino acid metabolism, citrate cycle, and protein folding modules.

## Discussion

This study was undertaken to predict the bacterial functions in the CVM of South African women with and without BV and HR-HPV infection. The structure of the female genital tract provides a unique milieu for growth of endogenous *Lactobacillus* spp. that promote cervicovaginal health by reducing the risk of STIs through intrinsic mechanisms [1]. However, little is known about the functional composition of the CVM and how it varies by cervicovaginal syndromes, infections, and diseases. By using V3-V4 16S rRNA gene metabarcoding and computational approaches, we observed that women with and without high-diversity CVM, BV, and HR-HPV have different functional composition.

We identified eight CVM clusters (CSTs) in reproductive-age South African women. Three of these CSTs were dominated by common *Lactobacillus* spp. (CST-1: *L*. *crispatus*, CST-2: *L*. *jensenii*, or CST-3: *L*. *iners*), four by less frequently reported non-lactobacilli (CST-4: *Aerococcus* sp., CST-5: *Streptococcus* sp., CST-6: *C*. *trachomatis*, or CST-7: *Corynebacterium* sp.), and one by a mixture of BV-associated bacteria (CST-8). These CSTs are discussed in detail elsewhere [30]. Of the most frequently isolated *Lactobacillus* spp. from the cervicovaginal milieu, *L*. *crispatus* and *L*. *iners* are thought to have the highest and lowest protective functional values, correspondingly [1,9]. Analogous to previous reports of African women [2,4,6–8], we noted that CST-3 and CST-8 were the most prevalent in our study population. CST-8 was more diverse than CST-3 and was associated with BV. This finding was not unexpected since it has been corroborated in previous reports [6,8,13] and that non-*Lactobacillus* CVM may facilitate transition to dysbiotic CVM or BV state [1]. The alpha diversity of the CSTs as captured by the rank abundance plots, reiterated our previous observations using classical measures of alpha diversity, including Shannon diversity index [30]. Because of the very low frequency of CST-1, CST-2 and CSTs 4–7, only CST-3 and CST-8 were included in the predictive functional profiling analyses. Apart from *L*. *iners* (mean proportion: 82.4% versus 2.6%), *L*. *crispatus*, albeit in low proportion (1.2% versus 0.002%), was more abundant in CST-3 compared to CST-8. Higher proportions of BV associated bacteria such as *G*. *vaginalis* (12.7% versus 3.1%), *A*. *vaginae* (4.4% versus 0.2%), and *Megasphaera* sp. (12.3% versus 0.7%) differentiated CST-8 from CST-3. Although not investigated, we hypothesize that such differentially abundant bacteria and their multipartite relationships with one another could be responsible for the differences in the inferred metagenomic functional profiles between the two CSTs.

We compared the relative abundances of the predicted metagenome functions of CVM in CST-3 and CST-8, and CVM of women with and without BV and HR-HPV infection. Several differences in inferred KEGG functional categories that have been previously identified in cervicovaginal and uterine microbiota [21,23–25], were found in all three comparisons; thus, suggesting a connection between CVM, BV, and HR-HPV-infection. Interestingly, a majority of the predicted functional categories including, but not limited to, sporulation, bacterial motility proteins, flagellar assembly, peroxisome, and ion channels, were consistent with those observed among Taiwanese women with and without BV as diagnosed by both Amsel’s criteria and Nugent scoring [21]. Moreover, the observed clear separation of the inferred functional categories on PCoA according to BV status is analogous to the Taiwanese study, only in the case where the investigators diagnosed BV using the Nugent scoring system [21]. Functional diversity has been observed in CVM [25]. Some of the inferred functional modules (e.g., energy metabolism, DNA repair, and cell envelope biogenesis) have been underscored in the pangenome of *L*. *crispatus* [42]. In our study, we mostly focused on the most significant differences in predicted functional categories between the comparison groups. It is also important to note that the abundances of the bacterial functions are extrapolated from the abundances in the 16S rRNA data. The functions found to be significantly enriched in each comparison group, therefore, provides insight into the genomic capacities of the predominant bacterial taxa within that group. Transporters, including ABC transporters, which have been reported in cervicovaginal samples [23], were strongly associated in CST-8. ABC transporters are membrane proteins that facilitate the transport of a variety of substrates across the bacterial membrane, ranging from sugars, amino acids to xenobiotic. The ability to facilitate the uptake and export of a wide range of substrates enables their involvement in several cellular processes such as nutrient uptake, secretion of cellular waste, osmotic stress, lipid transport and macromolecular transport during biogenesis [43]. In the genitourinary system, they may be involved in urinary tract infection [44] and drug disposition such as regulating the effect of antiretroviral drugs [45]. ABC transporters are also known to contribute to antimicrobial drug resistance [46]. Genes encoding proteins involved in transport, including ABC-type transporters, are abundant in the genomes of many *G*. *vaginalis* and *Sneathia* sp. strains [47,48]. In our cohort, these bacteria were predominant in CST-8 and BV-positive CVM and have been associated with HR-HPV infection [30] and its progression to CIN2+ [28].

*G*. *vaginalis* is thought to be the driver of community diversity and biofilm development [49]. In CST-8 (a highly diverse CVM), which was associated with BV, *G*. *vaginalis* co-occurred with BV-associated bacteria. Interestingly, it has been reported that *G*. *vaginalis* associated with BV and biofilms uniquely encode genes for ABC transporters that are absent in the genomes of *G*. *vaginalis* strains not associated with BV [50]. Furthermore, transcripts encoding ABC transporters are upregulated in *G*. *vaginalis* biofilm cells compared to planktonic cells [51]. Cells in biofilms are likely to encounter restricted availability of nutrients and the elevated levels of ABC transporter proteins may facilitate greater nutrient uptake and survival under these conditions. Solano and colleagues (2014) [52] stated that the dispersion of a mature biofilm, possibly regulated by quorum sensing, is vital for colonization of new niches by bacteria when their nutrients become depleted and waste products accumulate. Full colonization of niches by BV-associated bacteria in CST-8 may be enhanced through swarming motility, driven by quorum sensing. Flagellar assembly, bacterial chemotaxis, and bacterial motility proteins were enriched in CST-8. These may be used by bacteria in CST-8 to enhance their survival and/or cause dysbiosis. Published literatures underscore that chemotaxis is important for initial host infection and pathogenicity [29], that is, for directing the flagellar motility to the site of pathogenesis. Flagellar-mediated motility is crucial for expediting bacterial infection, invasion through self-induced phagocytosis, bacterial penetration between cell-cell junctions, and post-infection dispersal [53]. Moreover, it aids in the differentiation of bacterial planktonic form into mature biofilm [53–55]. ABC transporters are also involved in the shuttling of non-glycogen polysaccharides for metabolism and export of cell-surface glycoconjugates for biofilm formation and LPS O-antigen synthesis [56,57].

We further noted that fructose and mannose metabolic functionalities were enriched in CST-8, and CVM of women with BV and HR-HPV infection. Starch and sucrose metabolisms as well as PTS were additionally enriched in the CVM of women with BV. Specific PTS involved in pathway of starch and sucrose metabolism have been identified in cervical samples [25]. Fructose and starch are less abundant carbohydrate sources in the vagina [48]. *G*. *vaginalis*, a predominant member of CVM in CST-8, BV-positive and HR-HPV-positive women in our cohort [30], has the ability to metabolize both fructose and starch [48]. *L*. *iners*, the predominant member of CST-3 CVM, in contrast, does not have the genetic capacity to ferment fructose [58]. In a comparison of metabolites in cervicovaginal lavage fluid from 40 women with BV and 20 women without BV, fructose was found to be significantly lower in BV-positive samples [19]. This may be due to its metabolism by *G*. *vaginalis*. These findings are supported by a metaproteomic study that noted that proteins involved in catabolism and membrane transport functions were more abundant in CVM dominated with *G*. *vaginalis* than *L*. *iners* [18]. Examples of differentially abundant proteins included MalE-type ABC sugar transport system periplasmic component, α-1,4-glucan phosphorylase (an enzyme that degrades starch and glycogen), and fructose-1,6-bisphosphate aldolase (an enzyme catalyzing the reversible conversion of fructose 1,6-bisphosphate into triose phosphates). It can be argued that these significant enrichments possibly enable *G*. *vaginalis* to outmatch lactobacilli competency for the uptake of extracellular saccharide. It is necessary to underscore that, differences in the inferred functional contents of CVM of women with and without HR-HPV infection could be due to significant differences in the women’s age and cervical cytology. This is because compositional and/or functional differences in CVM may be impacted by reproductive aging [59] and cervical cytology [20,25].

CVM composition and function have a profound effect on women’s reproductive health. Lower relative abundances of *Lactobacillus* have been associated with BV, cervicitis, obesity [10], and HPV infection [4,10]. We previously associated higher relative abundances of *G*. *vaginalis* and other BV-associated bacteria, which were predominant in CST-8, with HR-HPV infection [30]. Communities dominated by *G*. *vaginalis* from women without BV have been associated with proteomic signatures of disrupted cervicovaginal epithelial integrity [18]. It is thought that loss of epithelial integrity may allow HPV particles to enter and infect the basal cells [60]. Subsequently, HPV may use its oncoproteins (specifically E6 and E7) to trigger oncogenic cellular transformation through deregulation of cellular signalling pathways and deregulation of tumour suppressor genes [61]. In the present study, functional cell motility pathway was enriched in women without HR-HPV infection. This pathway may be protective against HR-HPV infection as it has been negatively associated with HR-HPV progression to CIN2+ [28]. Thus, our conjecture here is that the non-*Lactobacillus*-dominated CVM with BV-associated bacteria have altered bacterial functions that may subsequently disrupt the host protective mechanisms against invasion by pathogens and pathobionts.

### Limitations

We acknowledge that 16S rRNA gene metabarcoding approach can result in selection biases of certain bacterial taxa, this stemming from amplification and sequencing biases. Our results might not be fully reliable since computational gene annotations may be inaccurate [62]. It has been documented that the accuracy of PICRUSt is dependent on 16S rRNA copy number and accurate gene annotations [26]. It is also possible that we may have missed to capture all the bacterial functions using the KEGG database–since a considerable fraction of the metagenome, about 33%, is not sufficiently represented by reference genomes [11]. Nonetheless, we did not identify the specific OTUs that were contributing each of the bacterial functions. Our results on the inferred functional metagenomic capacity of the CVM as predicted using PICRUSt were not adjusted for potential confounders. For example, our predictions were not devoid of all genital syndromes, infections, and diseases that might have obscured the exact functions. In addition to this, we overlooked the reported impact of human host genetics on the functional trait of CVM [10]. Therefore, these limitations should be addressed in future studies. Overall, despite the cited limitations, our study demonstrates the benefits of using predictive analyses to infer the functional composition of CVM in healthy and dysbiotic health states.

## Conclusions

In our cohort, the functional potential of CVM was impacted by microbial diversity and BV, but not HR-HPV infection. Our analysis revealed functional potential signatures of high-diversity CVM, BV and HR-HPV infections. Such differentially abundant functional categories in CVM of women with and without microbial diversity, BV, and HR-HPV infection may have diagnostic, therapeutic, and prognostic applications. Predicted functional categories that were common in women with high-diversity CVM, BV, and HR-HPV infection suggest interconnectivity between CVM and these reproductive outcomes (BV and HR-HPV infection). Our findings are hypothesis-generating and warrant confirmation using functional studies.

## Supporting information

S1 Fig PICRUSt accuracy across cervicovaginal samples as shown by the weighted nearest sequenced taxon index (NSTI) scores. NSTI value describes the average branch length that separates each OTU in a sample, weighted by the relative abundance of that OTU in the sample. NSTI values range from 0 to 1, with a high value depicting greater distance to the closest sequenced relatives of the OTUs in each sample. A higher value could be as a result of unexplored diversity. A lower NSTI depicts a higher similarity to the closest sequenced taxon. From the bar charts, most of the samples had weighted NSTI values of between 0.07 and 0.15; thus, reflecting availability of reference genomes and relatively good quality of the PICRUSt predictions.
(TIF)

S2 Fig Beta diversity metric with principal component analysis (PCoA) clustering of the predicted metagenomic content. PCoA according to high-risk human papillomavirus (HR-HPV) status (Maya blue: HR-HPV-negative and Indian red: HR-HPV-positive). Each solid dot represents one cervicovaginal sample. The first three PCoA axes and the percentage variation explained by each indicated are shown (Axis 1: 64.08%, Axis 2: 12.06%, and Axis 3: 5.065%).
(TIF)

S1 Table Comparison of the demographic, sociobehavioural, and clinical characteristics of the women with cervical microbiota belonging to community state type-3 (CST-3) (*L*. *iners*-dominated) and CST-8 (diverse). (DOCX)
