# Supplementary figures and images for: Predictive functional analysis reveals inferred features unique to cervicovaginal microbiota of African women with bacterial vaginosis and high-risk human papillomavirus infection
Source: PLoS One. 2021 Jun 18;16(6):e0253218. doi: 10.1371/journal.pone.0253218 (PMC8213166; doi:10.1371/journal.pone.0253218)

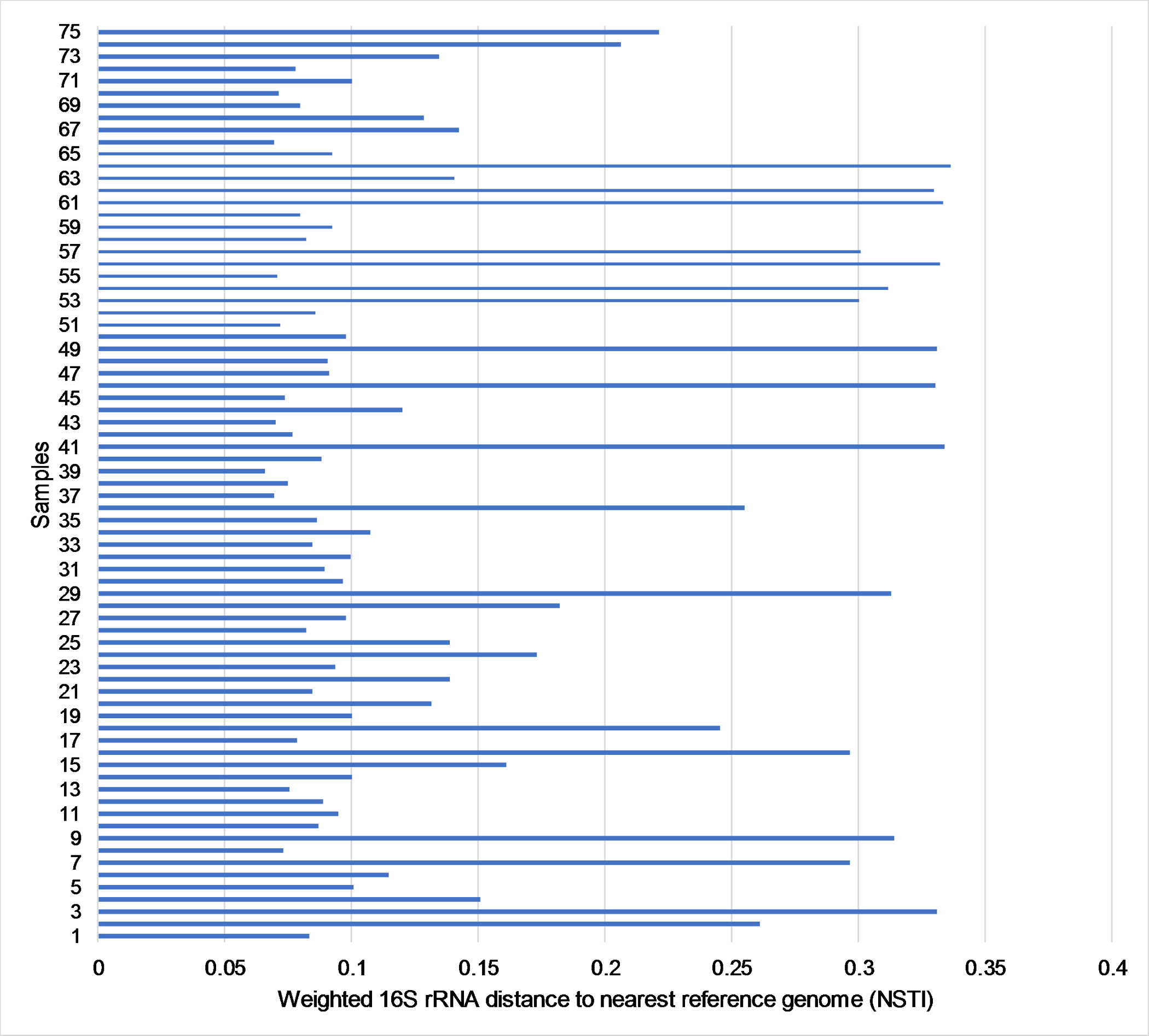

Supplement: S1 Fig — NSTI value describes the average branch length that separates each OTU in a sample, weighted by the relative abundance of that OTU in the sample. NSTI values range from 0 to 1, with a high value depicting greater distance to the closest sequenced relatives of the OTUs in each sample. A higher value could be as a result of unexplored diversity. A lower NSTI depicts a higher similarity to the closest sequenced taxon. From the bar charts, most of the samples had weighted NSTI values of between 0.07 and 0.15; thus, reflecting availability of reference genomes and relatively good quality of the PICRUSt predictions. (TIF) [file pone.0253218.s001.tif]

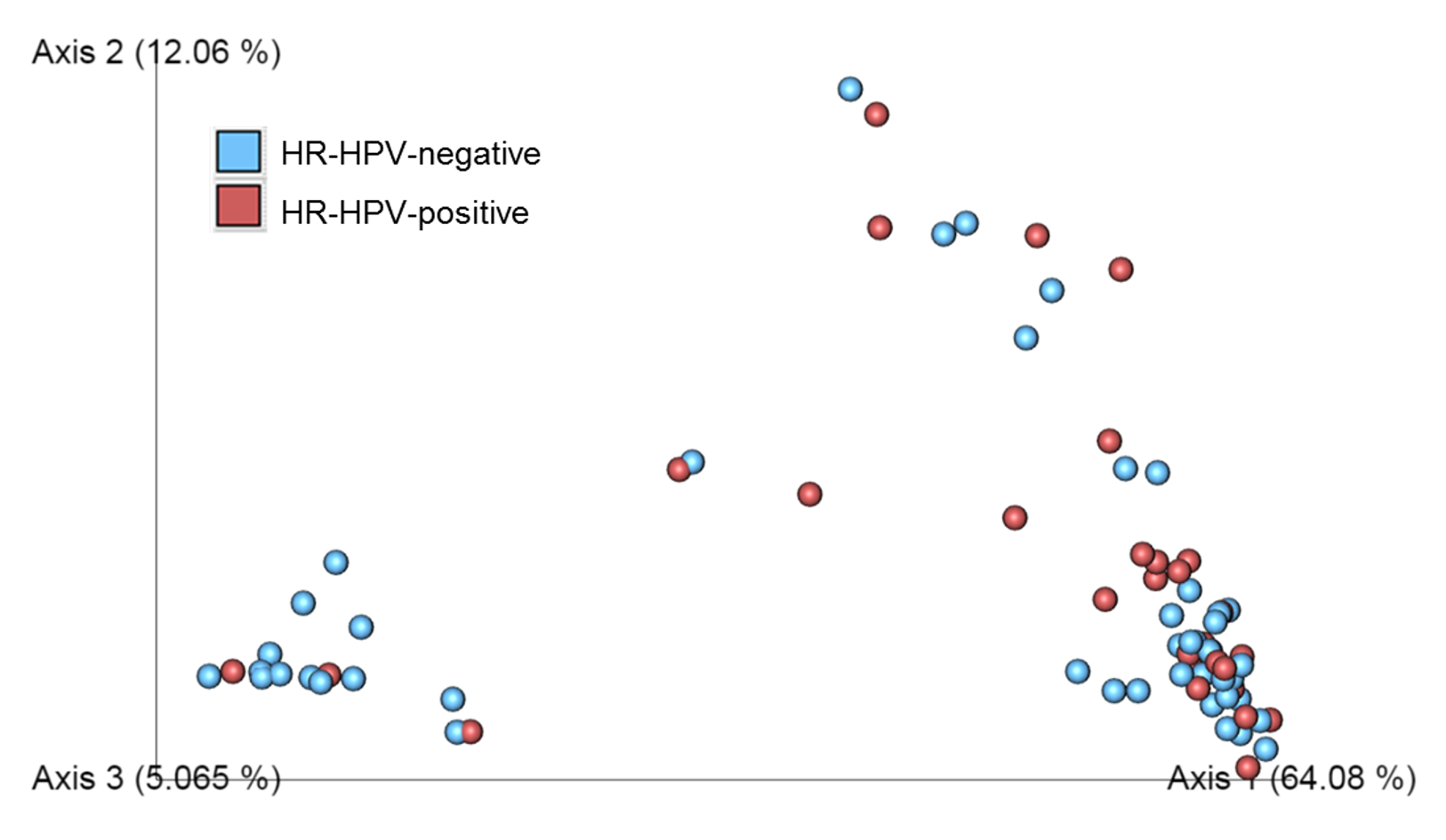

Supplement: S2 Fig — PCoA according to high-risk human papillomavirus (HR-HPV) status (Maya blue: HR-HPV-negative and Indian red: HR-HPV-positive). Each solid dot represents one cervicovaginal sample. The first three PCoA axes and the percentage variation explained by each indicated are shown (Axis 1: 64.08%, Axis 2: 12.06%, and Axis 3: 5.065%). (TIF) [file pone.0253218.s002.tif]
