# Supplementary material for: Predictive functional analysis reveals inferred features unique to cervicovaginal microbiota of African women with bacterial vaginosis and high-risk human papillomavirus infection
Source: PLoS One. 2021 Jun 18;16(6):e0253218. doi: 10.1371/journal.pone.0253218 (PMC8213166; doi:10.1371/journal.pone.0253218)
Supplement: S1 Table — (DOCX) [file pone.0253218.s003.docx]

**S1 Table. Comparison of the demographic, sociobehavioural, and clinical characteristics of the women with cervical microbiota belonging to community state type-3 (CST-3) (*L. iners*-dominated)** **and CST-8 (diverse).**

| Characteristic | | CST-3 | CST-8 | p*-*value^#^ |
| --- | --- | --- | --- | --- |
|  |  | **(N = 19, 25.3%)** | **(N = 56, 74.7%)** |  |
| **Age (years)** | | 29.0 (23.0-40.0) | 32.5 (25.3-39.0) | 0.531 |
| **HPV infection (% (n/N))** | |  |  |  |
|  | Any HPV type | 42.1 (8/19) | 50.0 (28/56) | 0.552 |
|  | Any high-risk HPV type | 26.3 (5/19) | 42.9 (24/56) | 0.201 |
|  | Single HPV infection | 15.8 (3/19) | 26.8 (15/56) | 0.625 |
|  | Multiple HPV infection | 26.3 (5/19) | 23.2 (13/56) |  |
| **HPV status at 6-month visit (% (n/N))** | |  |  |  |
|  | Negative | 56.3 (9/16) | 38.2 (13/34) | 0.501 |
|  | Acquired | 12.5 (2/16) | 11.8 (4/34) |  |
|  | Cleared | 0.0 (0/16) | 11.8 (4/34) |  |
|  | Persisted | 6.3 (1/16) | 2.9 (1/34) |  |
|  | Complex^+^ | 25.0 (4/16) | 35.3 (12/34) |  |
| **Age at sexual debut (years)^^^** | | 17.0 (15.0-19.0) | 17.0 (16.0-18.0) | 0.768 |
| **Lifetime number of sexual partners^^^** | | 3.0 (2.0-5.0) | 3.0 (2.0-4.0) | 0.998 |
| **Number of sex acts with study partner in last month^^^** | | 4.0 (2.0-7.0) | 4.0 (2.0-8.0) | 0.397 |
| **Current use of hormonal contraceptives* (% (n/N))** | | 44.4 (8/18) | 32.6 (15/46) | 0.375 |
| **Cervical cytology (% (n/N))** | |  |  |  |
|  | Normal | 77.8 (14/18) | 73.1 (38/52) | 0.051 |
|  | ASCUS | 0.0 (0/18) | 19.2 (10/52) |  |
|  | LSIL | 22.2 (4/18) | 7.7 (4/52) |  |
|  | HSIL | 0.0 (0/18) | 0.0 (0/52) |  |
| **Experienced vaginal discharge in last 6 months (% (n/N))** | | 10.5 (2/19) | 16.0 (8/50) | 0.715 |
| **Incidental BV on Papanicolaou smear (% (n/N))** | | 0.0 (0/19) | 62.5 (35/56) | **<0.0001** |
| **Sexually transmitted infections** | |  |  |  |
| Chlamydia^&^ | | 42.1 (8/19) | 41.1 (23/56) | 0.937 |
| Trichomoniasis^&^ | | 10.5 (2/19) | 5.4 (3/56) | 0.596 |
| **Cigarette use (% (n/N))** | |  |  |  |
|  | Never smoked | 63.2 (12/19) | 64.3 (36/56) | 0.949 |
|  | Ex-smoker | 5.3 (1/19) | 3.6 (2/56) |  |
|  | Current smoker | 31.6 (6/19) | 32.1 (18/56) |  |

Abbreviations: HPV – human papillomavirus, ASCUS – atypical cells of undetermined significance, LSIL – low-grade squamous intraepithelial lesion, HSIL – high-grade squamous intraepithelial lesion, BV – bacterial vaginosis, CST-3 - *L. iners*-dominated cervicovaginal microbiota, CST-8 – diverse cervicovaginal microbiota.

^#^p-values are shown for comparison of each variable between CST-3 and CST-8. Associations of continuous variables (expressed as medians with interquartile ranges (IQRs, at 25^th^ and 75^th^ percentiles)) and categorical variables were computed by Mann-Whitney unpaired and Chi-square/Fisher’s exact tests, respectively. Significant p-values (<0.05) are shown in **bold**.

^Data was not available on the age at sexual debut for one woman (CST-8), number of lifetime sexual partner for one woman (CST-8), and number of sexual acts with study partner in the last month for two women (CST-8).

^+^Women who had various combinations of acquired, cleared and/or persistent HPV infections at the 6-month follow-up.

*The hormonal contraceptives included oral pills, norethisterone enanthate, Depo-Provera, and steroids.

^+^The “complex” HPV status consisted of women that had cleared one HPV genotype but were infected with new genotypes and/or had other genotypes that had persisted at the 6-month follow-up.

^&^Chlamydia was detected by deep sequencing while trichomoniasis screened by wet microscopy.
